# Primary HSV-2 Infection in Early Pregnancy Results in Transplacental Viral Transmission and Dose-Dependent Adverse Pregnancy Outcomes in a Novel Mouse Model

**DOI:** 10.3390/v13101929

**Published:** 2021-09-25

**Authors:** Allison M. Felker, Philip Nguyen, Charu Kaushic

**Affiliations:** 1 McMaster Immunology Research Centre, Michael G. DeGroote Centre for Learning and Discovery, McMaster University, Hamilton, ON L8S 4L8, Canada; Philip.nguyen2@griffithuni.edu.au; 2 Department of Medicine, McMaster University, Hamilton, ON L8S 4L8, Canada

**Keywords:** HSV-2, viral infection, pregnancy, placental pathology, neonatal herpes, mouse model

## Abstract

Herpes simplex virus type 2 (HSV-2) infection affects 24 million births annually and is associated with adverse pregnancy outcomes, including neonatal herpes; however, the mechanisms underlying in utero transmission of HSV-2 are largely unknown. We examined the effects of primary HSV-2 infection during early pregnancy on gestational outcomes in a novel, clinically relevant mouse model. Pregnant C57BL/6 mice were infected intravaginally with 10^2^–10^5^ pfu/mL HSV-2 on gestation day (gd) 4.5. Controls were infected, nonpregnant, diestrus-staged mice and pregnant, uninfected mice. Compared to nonpregnant mice, pregnant mice were 100-fold more susceptible to HSV-2 infection. Three days post-inoculation (gd7.5), viral DNA was present in implantation sites, but pregnancy outcomes were largely unaffected by infection. Eight days post-inoculation (gd12.5), HSV-2 DNA persisted in placental tissues, resulting in inflammation and hemorrhage. Fetal and placental weights were reduced and fetal loss was observed with high viral doses. HSV-2 DNA and increased expression of pro-inflammatory mediators were detected in fetal tissues at gd12.5, signifying viral transmission and fetal infection, even with low viral doses. This mouse model shows a dose-dependent effect of primary HSV-2 infection on pregnancy outcomes and suggests that fetal loss may occur due to placental inflammation, thus providing valuable insight into in utero transmission of HSV-2.

## 1. Introduction

Herpes simplex virus type 2 (HSV-2) is a double stranded DNA virus of the *Herpesviridae* family. It is the primary cause of genital herpes and is one of the most common sexually transmitted infections. Approximately 491.5 million people, or 13.2% of the world’s population aged 15–49, are living with HSV-2 and 23.9 million new cases are reported each year [1]. Global prevalence of HSV-2 is astonishingly high, as the majority of HSV-2 cases are subclinical and asymptomatic viral shedding results in high rates of viral transmission. In areas where HSV-2 is endemic, women of reproductive age have the highest incidence of HSV infection, with approximately 313.5 million women infected globally, compared 178.0 million men [1]. Part of this imbalance is attributed to the elevated susceptibility of the female reproductive tract (FRT) to HSV-2, although the exact mechanisms have not been elucidated exhaustively. Results from mouse models indicate that the female sex hormone, progesterone, which is high in the diestrus phase of the mouse estrous cycle, increases HSV-2 susceptibility in the FRT through modulation of the epithelial cell barrier and immune function [2,3,4]. Subsequent in vitro studies utilizing a human vaginal epithelial cell line (Vk2/E6E7) further demonstrated that progesterone exposure confers higher susceptibility to HSV-2 infection [5].

During pregnancy, the risk of acquiring HSV-2 (primary infection), and the risk of a recurrence in women previously infected with the virus, is heightened [6,7,8]. Maternal infection with HSV-2 can occur at any stage of pregnancy, and, regardless of whether the infection is recurrent or acquired during pregnancy, it can be transmitted to the fetus, resulting in neonatal herpes infection [9,10]. Globally, it is estimated that 24 million women with either preexisting or primary HSV-2 infection give birth every year [11]. In the United States, approximately 22% of pregnant women are seropositive for HSV-2 and a further 2–3% had a primary HSV-2 infection during pregnancy [6,12]. Among women seropositive for HSV-2, approximately 75% will have at least one recurrence of active viral infection before the third trimester [12,13] and 14% will have prodromal symptoms or clinical recurrence at the time of delivery [13]. Compared with uninfected pregnancies, intrauterine HSV-2 infection is linked to numerous adverse pregnancy outcomes including spontaneous abortion [14,15,16], stillbirth [9,14,17], intrauterine growth restriction (IUGR) [14,18,19], low birth weight [16,18], and preterm birth [7,14,16,18,20].

Of the numerous maternal and fetal complications associated with HSV-2 infection during pregnancy, viral transmission to and acquisition of HSV-2 by the neonate is perhaps the most devastating outcome. Neonatal HSV infection has several clinical manifestations: skin, eyes, and mouth herpes (SEM) disease, central nervous system (CNS) disease, and disseminated disease, the most severe form of neonatal infection [10]. Neonatal herpes carries a mortality rate of approximately 60% when left untreated [11] and around 20% of neonatal herpes survivors will have long-term neurologic sequelae [13]. Estimates from the United States suggest that approximately 1200–1500 cases of neonatal herpes occur annually [13]; globally, the estimated incidence of neonatal herpes infection is 10.3 per 100,000 live births [6]. Prevention of neonatal transmission remains a challenge and depends primarily on the prevention of maternal infection during the antenatal, intrapartum and postnatal periods. In most instances (85% of cases), neonatal HSV-2 acquisition occurs at the time of delivery during the peripartum period. Cesarean delivery can minimize risk, but it does not completely prevent neonatal exposure [13]. While considerably rarer (1 in 300,000 live births in the United States) [6], vertical transmission of HSV-2 in utero (5% of neonatal herpes cases) results in a more severe form of congenital HSV-2 infection [11,12,14,21], which is associated with hydrocephalus, intracranial calcifications, and microcephaly [9], and poses a high risk of neonatal morbidity and mortality. Pregnancies in women with recurrent HSV-2 infection carry a low risk of neonatal or congenital herpes infection due to the presence of protective maternal IgG antibodies and the availability of antiviral therapies [9,13,22]. However, a recent study suggests that even in asymptomatic women, HSV-2 is detectable in fetal placental tissues at term [23], and antiviral treatment does not completely prevent perinatal or in utero transmission to the neonate [6,9]. Comparatively, in women who acquire HSV-2 during pregnancy (primary maternal infection), the risk of viral transmission to the neonate in utero is considerably higher [9] and maternal infection is usually asymptomatic [24]. Since current recommendations from the Centers for Disease Control, U.S. Preventive Services Task Force, and American and Royal Colleges of Obstetricians and Gynecologists do not support screening for HSV infection in asymptomatic pregnant women [6,25,26], many of these infections go unnoticed and untreated. Therefore, it is not surprising that in pregnancies complicated by primary maternal HSV-2 infection, an estimated 40–80% will lead to neonatal herpes infection, resulting in high rates of perinatal and infant mortality [11,13,14].

Despite the high prevalence of HSV-2 among pregnant women and the global burden associated with maternal and/or fetal morbidities due to HSV-2 infection, very little is known about the mechanisms of HSV-2 infection and transplacental viral dissemination during pregnancy. Contributing to this lack of understanding is the lack of a relevant animal model of HSV-2 infection during pregnancy. In one study by Sanjuan et al., the authors intravaginally (IVAG) inoculated BALB/C mice with a high dose of HSV-2 (5 × 10^5^ plaque forming units (pfu)) during two timepoints in early and mid-pregnancy. Following inoculation, they observed high rates of maternal mortality and fetal resorption in infected animals [27]. However, this study used a high viral dose previously shown to be lethal in nonpregnant mice inoculated in the high-progesterone, diestrus stage of the mouse estrous cycle [2]. Moreover, no follow up studies were performed to investigate the preliminary fetal loss observed in this model. Similarly, a recent study by LaTourette et al. investigated the impact of pre-pregnancy vaccination in female mice for protection against neonatal herpes in pups. In their model, pups were infected with HSV-2 on postnatal day 3 and assessed for neutralizing antibodies following infection [28]. While this study demonstrates the importance of maternal immunization for neonates, it does not address the consequences of untreated maternal HSV-2 infection during pregnancy, including in utero viral transmission. As such, the need for a clinically relevant mouse model of HSV-2 in pregnancy remains unmet. In this study, we developed a mouse model of HSV-2 infection during early pregnancy to examine the effect of low and high doses of HSV-2 on pregnancy outcomes. We further aimed to characterize potential mechanisms of fetal loss through investigation of systemic viral dissemination and histopathological analysis of placental tissues.

## 2. Materials and Methods

### 2.1. Mice

Inbred, 6–8-week-old virgin female C57BL/6 mice were obtained from Charles River Laboratories (St-Constant, QC, Canada). Mice were housed in the Central Animal Facility at McMaster University under a 12 h light/dark cycle and provided food and water *ad libitum.* Mice were mated by overnight cohabitation with syngeneic C57BL/6 males. Pregnancies were timed from detection of a copulation plug, considered gestation day (gd)0.5; euthanasia was by cervical dislocation at gd7.5 or gd12.5. Female nonpregnant control C57BL/6 mice were cycle staged for diestrus, when mice are known to be susceptible to HSV-2 infection [2,3] and progesterone levels are at their peak, and thus, most closely emulate the progesterone-dominant hormone milieu of pregnancy [29]. Mice were cycle staged by pipetting 30 μL of phosphate buffered saline (PBS) into and out of the vagina 5–6 times and observing cell populations by light microscopy. Mice with vaginal smears showing approximately 80% leukocytes and few epithelial cells were considered to be in diestrus. 

### 2.2. Primary HSV-2 Inoculation

Nonpregnant, diestrus-staged mice or pregnant, gd4.5 mice were anesthetized using an injectable anesthetic (150 mg of Ketamine^®^/kg (Bimedia-MTC, Cambridge, ON, Canada) with 10 mg of Xylazine^®^/kg (Bayer, Toronto, ON, Canada)) delivered intraperitoneally at a dose of 0.1 mL/10g body weight. Prior to inoculation, anesthetized mice were gently swabbed IVAG with sterile, dry cotton wool. Anaesthetized mice were inoculated IVAG with 10 μL of 10^2^, 10^3^, 10^4^, or 10^5^ pfu/mL wild type (WT) HSV-2 strain 333. This is consistent with doses utilized in previously published protocols, where diestrus-staged mice were consistently infected with 10^5^ pfu/mL HSV-2, and inoculation doses as low as 10^2^ pfu/mL could establish infection in mice treated with exogenous hormones [2,3]. After inoculation, mice were placed on their backs for approximately 30–45 min to allow the virus to infect the vaginal tract. Additional pregnant mice (normal controls) were anesthetized as above but were not inoculated with HSV-2 to account for the potential impact of the injectable anesthetic on pregnancy outcomes [30].

### 2.3. Vaginal Washes and Pathology Scoring

Vaginal washes were collected daily for up to 8 consecutive days following HSV-2 inoculation. Washes were collected by pipetting 30 μL of PBS twice into and out of the vagina 5–6 times. Samples were stored at −80 °C until use. Genital pathology was monitored daily following HSV-2 infection and was scored on a 5-point scale: 0, no infection; 1, slight redness of external vagina; 2, swelling and redness of external vagina; 3, severe swelling and redness of both vagina and surrounding tissue and hair loss in genital area; 4, genital ulceration with severe redness; 5, severe genital ulceration extending to surrounding tissue or hind limb paralysis [2,31,32]. Mice were euthanized by cervical dislocation when they reached a pathology score of 4 or 5. In order to compare groups, cumulative scores of pathology were determined by tabulating the number of mice with the highest score of pathology they achieved and the number of days that score was observed, as performed previously [31,32]. Mice that did not survive to the end of the challenge were given the highest pathology score at the time of death and assigned that score for each day that remained in the duration of the experiment. In this way, overall pathology was accurately reported for each group. The sum of all the scores for all the mice in each group was the total level of pathology for that group and then the average pathology score per mouse for each group was calculated by dividing total pathology by the number of mice in each group. Survival analysis was conducted up to 16 days post inoculation and included 12–16, nonpregnant, diestrus-staged mice, and 7–17 pregnant mice in each viral dose group. Daily pathology was monitored up to 8 days post inoculation and included 12–16 nonpregnant, diestrus-staged mice, and 7–17 pregnant mice in each viral dose group. Cumulative pathology scoring was calculated for mice with confirmed, productive infection 8 days post inoculation and included 6–8 nonpregnant, diestrus-staged mice and 3-8 pregnant mice in each viral dose group.

### 2.4. Viral Titers

Viral titers in vaginal washes were determined using a Vero cell (obtained from ATCC, Manassas, VA, USA) viral plaque assay. Briefly, Vero cells were grown to confluence in 12-well plates in α-Minimum Essential Medium (α-MEM) (Gibco Laboratories, Burlington, ON, Canada) supplemented with 5% fetal bovine serum (FBS; Gibco Laboratories), 1% penicillin-streptomycin (Invitrogen, Burlington, ON, Canada), L-glutamate (BioShop Canada Inc., Burlington, ON, Canada) and 1% HEPES (Invitrogen). Vaginal washes were thawed on ice, diluted (10^−2^ to 10^−7^) in FBS-free α-MEM and added to Vero cell monolayers. After 2 h incubation at 37 °C, monolayers were overlaid with 1.5 mL 5% FBS α-MEM to stop cellular adsorption of virus. After 48 h at 37 °C, cells were fixed and stained with Crystal Violet (Sigma-Aldrich, Oakville, ON, Canada) and viral plaques were enumerated under an inverted light microscope. Viral titers were calculated as pfu/mL using plaque counts for every sample and the relevant dilution factor. Viral titration assays were conducted up to 6 days post inoculation and included 3–8 nonpregnant, diestrus-staged mice and 5–7 pregnant mice in each viral dose group.

### 2.5. Viral DNA Extraction

At the time of sacrifice (gd7.5 or gd12.5), mice were dissected and the vagina and implantation sites were collected in RNAlater^®^ Solution (Sigma-Aldrich) and stored at −80 °C for viral DNA extraction. At gd12.5, when the placental structures are fully formed, the decidua, placenta and fetus were carefully dissected and stored as separate tissues; tissue weights were recorded prior to storage. Whole implantation sites were collected at gd7.5. To extract viral DNA, standard protocols from the DNeasy Blood & Tissue Kit were followed (Qiagen, Toronto, ON, Canada). First, 20 mg of tissue was finely minced in 180 μL of Buffer ATL followed by addition of 20 μL of proteinase K. The mixture was pulse vortexed for 15 s and incubated overnight at 56 °C. Following incubation, 200 μL of Buffer AL and 200 μL of anhydrous ethyl alcohol were added; the sample was thoroughly mixed by pulse-vortexing following each addition. The solution was added to a DNeasy Mini spin column and centrifuged at 8000 rpm for 1 min. Samples were washed with 500 μL Buffer AW1, centrifuged for 1 min at 8000 rpm, washed with 500 μL Buffer AW2, and centrifuged at 14,000 rpm for 3 min. Finally, samples were eluted in 100 μL of Buffer AE, twice, with centrifugation at 8000 rpm for 1 min. Following extraction, DNA yield and purity were assessed using a NanoVue Plus spectrophotometer (GE Life Sciences, Mississauga, ON, Canada) and products were stored at −20 °C.

### 2.6. Quantification of HSV-2 Viral DNA

Following DNA extraction, real-time quantitative PCR (qPCR) was performed using Applied Biosystems^™^ SYBR^™^ Select Master Mix (ThermoFisher Scientific). HSV-2 DNA was detected using previously published primer sets: F: 5′-GGG GTG ATC GGC GAG TAY TG-3′, R: 5′-ATC TGC TGG CCG TCG TAR ATG-3′ (Integrated DNA Technologies, Coralville, IA, USA) [33]. A standard curve was generated using known quantities of HSV-2 (G strain) Quantitated Viral DNA (08-922-000; Advanced Biotechnologies, Columbia, MD, USA). Each tissue sample was tested in triplicate and the number of viral copies per microliter was calculated from the standard curve. Samples were subjected to the following thermal conditions: 50 °C for 3 min, 95 °C for 2 min, followed by 40 cycles of 95 °C for 3 s, 60 °C for 30 s and 72 °C for 15 s. Data were collected and a melt curve was generated using an Applied Biosystems^TM^ StepOnePlus^TM^ Real-Time PCR System (ThermoFisher Scientific). Data were analyzed using StepOne^TM^ Software v2.3. Normal mice were included in qPCR experiments to serve as negative controls and demonstrate the validity of the qPCR protocol. DNA extraction and quantification was performed in triplicate and conducted using at least 2 implantation sites from 4–6 pregnant mice at gd7.5 and 3–5 pregnant mice at gd12.5 in normal control and viral dose groups.

### 2.7. Histology and Morphometry

Implantation sites and vaginal tracts from gd7.5 and gd12.5 mice were dissected, immersion-fixed in 10% formalin, processed, and paraffin embedded. Implantation sites from each pregnancy were cut as serial sections of 4 μm and mounted on Aptex-coated positive charge glass slides (Leica Biosystems, Concord, ON, Canada). Each staining procedure (outlined below) was conducted as replicate experiments. Stained slides were viewed and imaged using a Zeiss M2 Imager (Zeiss, Toronto, ON, Canada). All histological staining and morphometric quantifications were conducted using at least 2 implantation sites from 4–6 pregnant mice at gd7.5 and 3–5 pregnant mice at gd12.5 in normal control and viral dose groups.

#### 2.7.1. Hematoxylin and Eosin (H&E) Staining

After sectioning, slides containing implantation sites and vaginal tissue were deparaffinized, rehydrated, and stained using previously established protocols. Briefly, nuclei were stained with Modified Mayer’s Hematoxylin (Sigma-Aldrich, Oakville, ON, Canada) for 4 min and rinsed with running tap water. Subsequently, slides were dipped in weak Acid Alcohol (15 mL of 1% Acid Alcohol in 190 mL distilled water), rinsed with running tap water, and placed in Tris buffer pH 7.6 for 2 min. Next slides were placed in buffered 0.5% Eosin pH 5.5 for 5 min (Sigma-Aldrich), followed by running tap water and dehydration in increasing concentrations of ethanol. Slides were placed in xylene, mounted in Permount (Fisher Scientific, Ottawa, ON, Canada) and coverslipped. For arterial morphometric measurements, all spiral arteries in 2–3 sections per implantation site (n = at least 2 implantation sites per mouse and 3–6 animals per group) were assessed in images obtained at 100× magnification. A wall-lumen ratio for each artery was calculated using the total vessel and luminal areas in the following calculation: (A_Total_ − A_Lumen_)/A_Lumen_ and an average value per group was obtained [34]. Within the placental labyrinth, maternal and fetal vessels were identified by the presence of enucleated and nucleated red blood cells, respectively. Vessel lumens were outlined and area was calculated to provide vascular space measurements for 2–3 images per implantation site (n = at least 2 implantation sites per animal and 3–6 animals per group). All spiral artery and vascular space measurements were performed using Zen 2.5 Blue Software (Zeiss, Toronto, ON, Canada).

#### 2.7.2. HSV-2 Immunohistochemistry (IHC)

After sectioning, slides were deparaffinized, rehydrated, and treated by heat-activated antigen retrieval using citrate buffer (10 mM) pH 6.0 for 10 min. Slides were allowed to cool to room temperature, washed with PBS, and incubated in Hydrogen Peroxide Block from a Rabbit-specific HRP/DAB (ABC) Detection IHC Kit (ab64261; Abcam, Toronto, ON, Canada) for 10 min at room temperature to block endogenous peroxidase activity. After washing with PBS, sections were blocked with detection kit protein block reagent for 10 min at room temperature and subsequently incubated overnight at 4 °C with 1:200 biotinylated rabbit herpes simplex virus type 1/2 polyclonal antibody (PA1-7488; ThermoFisher Scientific, Waltham, MA, USA). Following primary antibody incubation, slides were washed with PBS and sections were covered with ExtrAvidin Peroxidase (1:200, E2886; Sigma-Aldrich) for 1 h at room temperature. After rinsing with PBS, slides were incubated in fresh 3,3′-Diaminobenzidine (DAB, 0.5 mg/mL, ab64238; Abcam), color change was observed under a light microscope, and the reaction was stopped by placing slides in distilled water. Slides were then counterstained with Harris’ Hematoxylin (Cedarlane, Burlington, ON, Canada) for 1 min and rinsed with running tap water. Slides were dehydrated, mounted in Permount (Fisher Scientific), and coverslipped

### 2.8. Tissue Homogenization and Multiplex Cytokine/Chemokine Analysis

At the time of sacrifice (gd12.5), implantation sites were removed and decidua, placenta and fetus were carefully dissected; tissue weights were recorded prior to storage. Individual tissues were placed in 500 µL PBS in sterile RINO^®^ tubes (TUBE1R5-S; FroggaBio, Toronto, ON, Canada) with a mixture of small and large stainless-steel beads. Tissues were homogenized for 5 min at 4 ℃ at maximum speed using a Bullet Blender^®^ Gold Homogenizer (NextAdvance, Troy, NY, USA). Following homogenization, samples were centrifuged at 8000 rpm for 5 min. Supernatants were collected and stored at −80 ℃ until future use. For cytokine/chemokine analysis, 80 µL of undiluted tissue homogenate supernatant was sent to Eve Technologies (Calgary, AB, Canada) for quantification using a mouse 31-plex array (MD31; Eve Technologies). Cytokine/chemokine quantification was performed using 2 implantation sites from 3 pregnant gd12.5 mice in normal, low dose (10^3^ pfu/mL) and high dose (10^5^ pfu/mL) groups.

### 2.9. Statistical Analysis

Statistical analysis was performed using GraphPad Prism 9.0 (GraphPad Software, San Diego, CA, USA). Data were analyzed for normality using the Shapiro–Wilk normality test. Infection rates were evaluated using Fisher’s exact test, differences in survival were calculated using the Mantel–Cox log rank test, and correlation between HSV-2 DNA and implantation site position was assessed using Spearman correlation coefficients. All other results were analyzed using one-way ANOVA with Tukey’s post-test. Significance was defined as *p* < 0.05 and data are expressed as means ± standard errors of the means (SEM).

## 3. Results

### 3.1. Pregnant Mice Are 100 Times More Susceptible to Primary HSV-2 Infection in Early Pregnancy than Nonpregnant, Diestrus-Staged Controls

**Figure 1:**
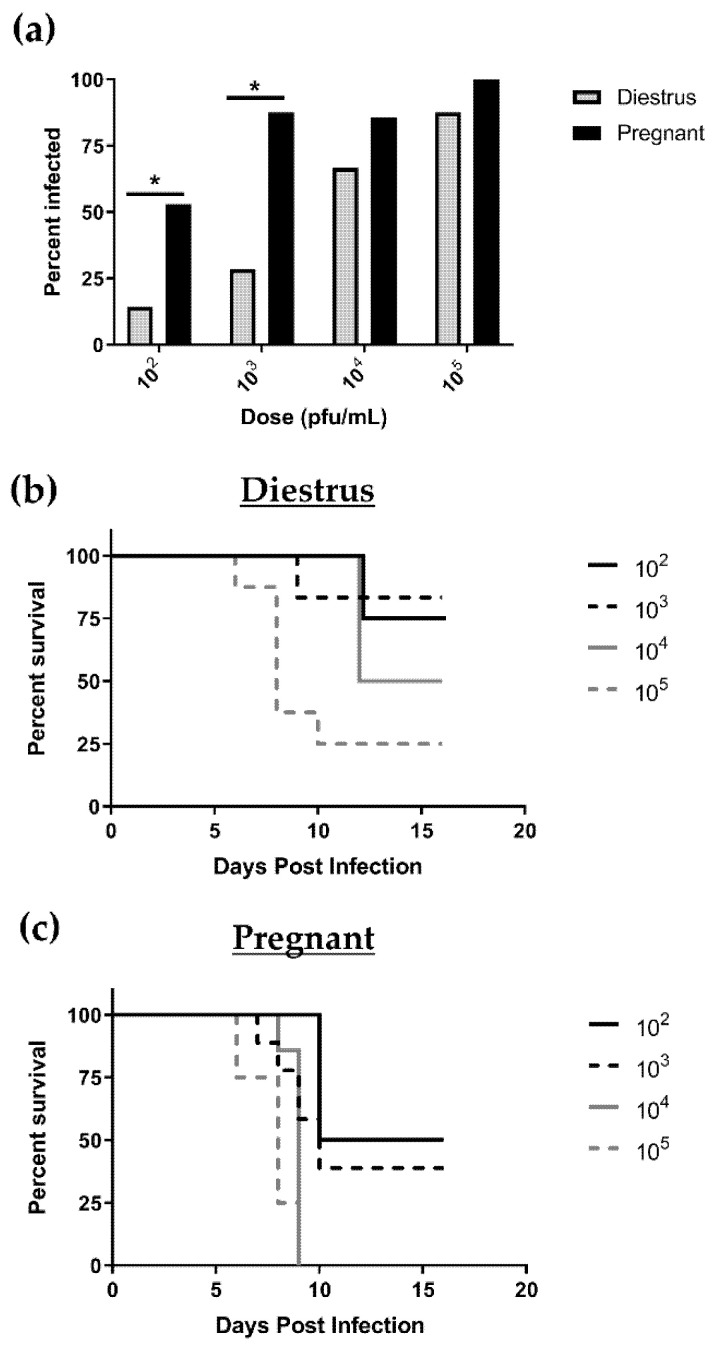
Pregnant mice are more susceptible to intravaginal HSV-2 infection than nonpregnant, diestrus-staged controls. (**a**) Following intravaginal inoculation with varying doses of HSV-2, productive infection was established in pregnant mice at greater rates than diestrus-staged controls, particularly at low viral doses of 10^2^ and 10^3^ pfu/mL. (**b**) Nonpregnant, diestrus-staged mice infected with HSV-2 were able to survive up to 16 days post infection (dpi). (**c**) HSV-2-infected, pregnant mice had decreased survival compared to diestrus-staged controls with 100% mortality observed by 9 dpi with high viral doses (10^4^ and 10^5^ pfu/mL). * *p* < 0.05.

Female C57BL/6 mice were mated to syngeneic males and the morning of copulation plug detection was deemed gd0.5. Mated females at gd4.5 and nonpregnant, diestrus-staged (progesterone high) control females were inoculated IVAG with HSV-2 at various doses (10^2^, 10^3^, 10^4^, or 10^5^ pfu/mL). Nonpregnant mice in the diestrus stage of the estrous cycle were used as controls since previous studies have shown that they are susceptible to HSV-2 at high doses [2,3]. The rate of productive viral infection at 3 or 8 days post-inoculation was assessed by genital pathology and viral shedding in vaginal washes. For all viral doses tested in both nonpregnant, diestrus-staged and pregnant mice, the rate of infection increased with increasing viral dose and was consistently higher in pregnant animals compared to nonpregnant controls (Figure 1a). This difference was more apparent with lower viral doses of 10^2^ pfu/mL, where 52.94% of pregnant mice were successfully infected compared with just 14.28% of nonpregnant, diestrus-staged mice (*p* = 0.0474) and 10^3^ pfu/mL (87.50% pregnant mice vs. 28.57% nonpregnant controls; *p* = 0.0201). At higher viral doses, the majority of animals in both groups showed evidence of productive infection at similar rates (10^4^ pfu/mL: 85.71% pregnant vs. 66.67% nonpregnant; *p* = 0.4902 and 10^5^ pfu/mL: 100% pregnant vs. 87.5% nonpregnant; *p* = 0.3329) (Figure 1a). Overall, the viral inoculation dose necessary to achieve 50% infection in pregnant mice was 100-fold lower compared to the inoculation dose that was necessary to achieve the same rate of infection in nonpregnant, diestrus-staged mice (10^2^ versus 10^4^ pfu/mL).

Next, survival percentages were assessed for HSV-2-infected mice in both groups up to 16 days post infection (dpi) (term pregnancy). Within the nonpregnant, diestrus-staged group, a proportion of mice survived to 16 dpi with all viral doses tested. Diestrus survival percentages ranged from 83% with 10^3^ pfu/mL to 25% with 10^5^ pfu/mL (Figure 1b). Pregnant animals fared significantly worse after HSV-2 infection, with only 39% of mice infected with 10^3^ pfu/mL surviving to 16 dpi and 100% of animals infected with 10^4^ and 10^5^ pfu/mL succumbing to infection by 9 dpi (Figure 1c). Similar to the rates of infection, a 100-fold increase in susceptibility of pregnant animals was observed in our survival data, where 50% mortality occurred at a viral dose of 10^4^ pfu/mL in nonpregnant, diestrus-staged controls compared to 10^2^ pfu/mL in pregnant mice.

### 3.2. HSV-2-Infected Pregnant Mice Display Higher Genital Pathology and Enhanced Viral Shedding Compared to Nonpregnant, Diestrus-staged Controls

Since pregnant mice were more susceptible to viral infection than nonpregnant controls, we next assessed the extent of HSV-2 infection in the genital tract up to 8 dpi, one day before 100% mortality was observed in pregnant mice infected with high viral doses. External genital pathology was monitored daily in infected nonpregnant and pregnant mice and scored on a 5-point scale, as described in the Materials and Methods. Mice were euthanized when they reached pathology scores ≥4. Mice were monitored for 8 dpi and data were expressed as individual pathology progression (Supplementary Figure S1) and cumulative pathology averaged per mouse for each treatment group (Table 1). Individual and cumulative pathology scores indicate that pathology increased with increasing viral dose in both nonpregnant, diestrus-staged and pregnant animals (Table 1, Supplementary Figure S1). The average pathology score for pregnant mice was significantly higher than nonpregnant, diestrus-staged controls at 10^2^ pfu/mL (2.8 vs. 0.7; *p* = 0.0228), 10^3^ pfu/mL (4.2 vs. 0.9; *p* = 0.0267), and 10^4^ pfu/mL (6.7 vs. 1.5; *p* = 0.0378), although pathology scores become comparable at the highest viral dose of 10^5^ pfu/mL (8.2 vs. 5.0; *p* = 0.1286) (Table 1). On average, pregnant mice began showing signs of pathology 1–2 days earlier than nonpregnant, diestrus-staged controls and disease progression was accelerated in pregnant mice (Supplementary Figure S1). Overall, following IVAG HSV-2 inoculation, local pathology scores were 4–5-fold higher in pregnant mice compared to nonpregnant, diestrus-staged controls.

**Figure 2:**
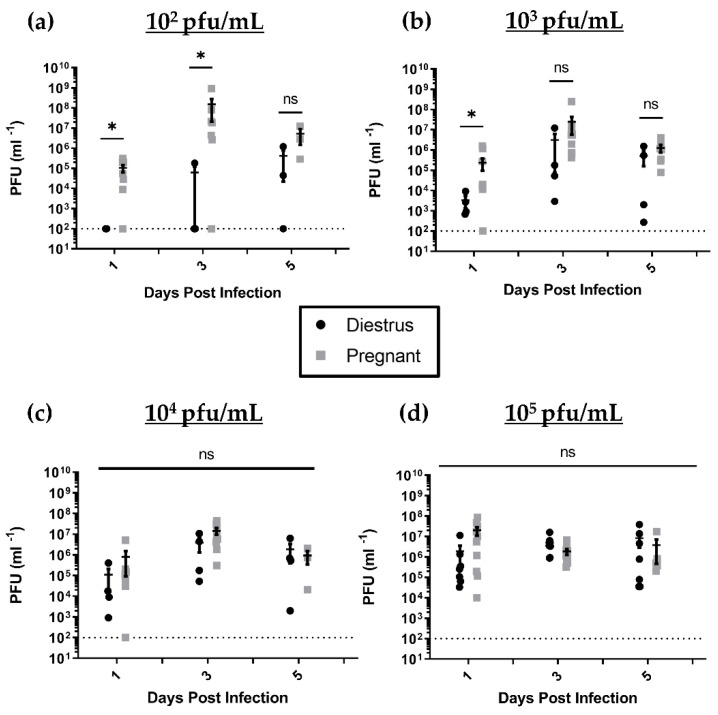
Viral shedding in vaginal washes collected from nonpregnant, diestrus-staged and pregnant mice at 1, 3 and 5 days post infection (dpi). (**a**) Following intravaginal (IVAG) infection with 10^2^ pfu/mL HSV-2, pregnant mice had increased viral shedding in vaginal washes collected at 1 and 3 dpi compared to diestrus controls. (**b**) At a viral dose of 10^3^ pfu/mL, higher rates of viral shedding were observed in pregnant mice compared to diestrus mice 1 dpi, but significant differences were absent at 3 and 5 dpi. In mice infected with high viral doses of (**c**) 10^4^ and (**d**) 10^5^ pfu/mL, there were no differences in viral titers in vaginal washes collected on any day. Data expressed as mean ± SEM. ns = not significant, * *p* < 0.05.

Viral shedding in nonpregnant, diestrus-staged mice and pregnant mice was assessed by viral plaque assay, as described in the Materials and Methods section, up to 6 dpi, after which time genital pathology necessitated cessation of sampling in highly infected animals. Mice inoculated with HSV-2 had high viral shedding in their vaginal washes for the first 6 days post-IVAG infection. In both nonpregnant, diestrus-staged and pregnant mice, viral shedding peaked at 3 dpi and declined towards 6 dpi (Figure 2). As such, data were analyzed further at 1, 3 and 5 dpi to assess viral progression (Figure 2). At low viral doses (10^2^ and 10^3^ pfu/mL), pregnant mice had consistently higher viral titers compared to nonpregnant mice. In pregnant mice inoculated with 10^2^ pfu/mL of HSV-2, there was significantly higher viral shedding than in nonpregnant, diestrus-staged controls at 1 (*p* = 0.0303) and 3 dpi (*p* = 0.0167) (Figure 2a). At a viral dose of 10^3^ pfu/mL, pregnant mice showed significantly elevated viral shedding at 1 dpi (*p* = 0.0061) compared to nonpregnant controls, but these differences were not sustained to 3 (*p* = 0.0777) or 5 dpi (*p* = 0.3152) (Figure 2b). At high viral doses (10^4^ and 10^5^ pfu/mL), nonpregnant and pregnant animals showed similar rates of viral shedding in vaginal washes at 1, 3 and 5 dpi (Figure 2c,d). Taken together, these results suggest that pregnant mice are more susceptible to HSV-2 infection, which spreads earlier and faster in these mice, leading to higher viral shedding at lower doses than nonpregnant, diestrus-staged control mice.

### 3.3. Following Primary Intravaginal Infection in Early Pregnancy, HSV-2 Ascends from the Vaginal Tract in a Directional Manner and HSV-2 DNA Is Detectable in Implantation Sites at gd7.5 and 12.5

Since the previous experiments confirmed the enhanced susceptibility of pregnant mice to HSV-2, we next examined the dissemination of HSV-2 in pregnant mice. Based on pathology and viral titers, two timepoints were selected for subsequent studies of HSV-2-infected, pregnant animals: 3 dpi corresponds with peak viral shedding in the vaginal tract and represents an early pregnancy timepoint (gd7.5) corresponding with trophoblast invasion into the maternal decidua [35]; 8 dpi is the final day that we saw survival in mice inoculated with 10^4^ and 10^5^ pfu/mL, and corresponds with gd12.5 of murine pregnancy after the placenta is fully developed [36].

**Figure 3:**
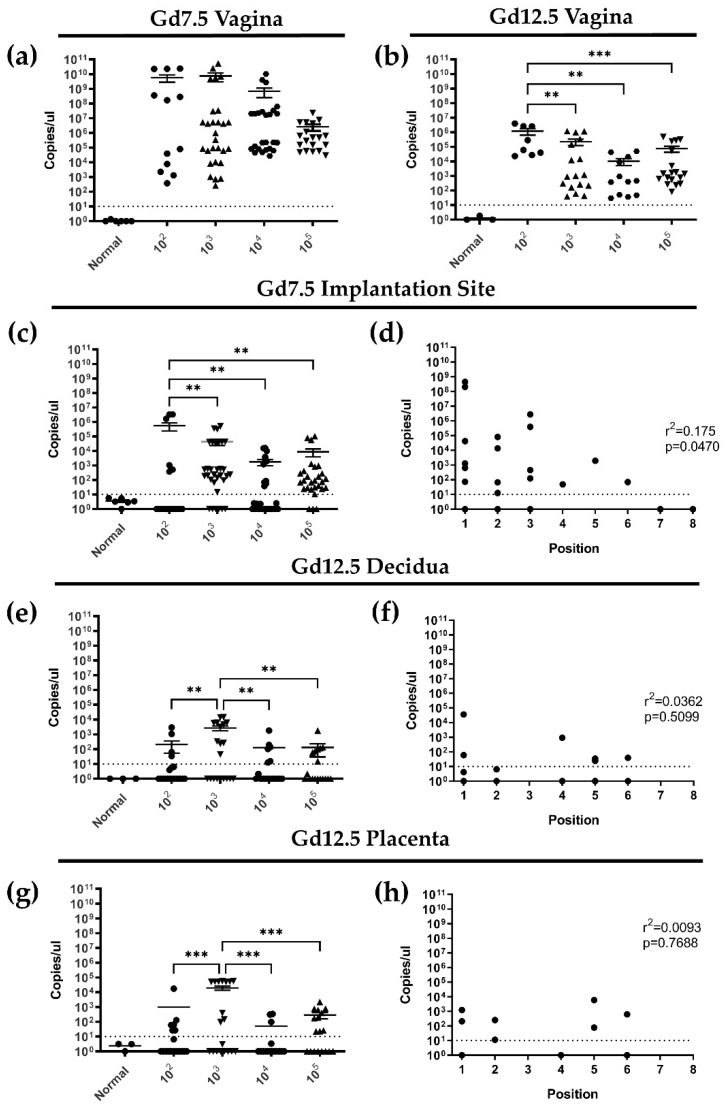
HSV-2 is detected in the vaginal tract by qPCR, ascends directionally into gd7.5 implantation sites, and persists in placental tissues 8 days post infection (dpi). (**a**) Quantification of HSV-2 DNA by qPCR revealed high viral loads within vaginal tissue at gd7.5 with all viral doses. (**b**) HSV-2 DNA persisted in vaginal tissue collected at gd12.5 but was detected at lower quantities than at gd7.5. (**c**) Quantification of HSV-2 DNA in gd7.5 implantation sites by qPCR. (**d**) HSV-2 viral load in gd7.5 implantation sites is dependent on location along the uterine horn, with higher copy numbers detected in implantation sites close to the cervix (position 1) as compared to those close to the ovary (position 8). (**e**) Quantification of HSV-2 DNA by qPCR in the decidua at gd12.5. (**f**) At gd12.5, HSV-2 viral load in the decidua is independent of implantation site position along the uterine horn. (**g**) Quantification of HSV-2 DNA by qPCR in the placenta at gd12.5. (**h**) HSV-2 viral load in the placenta is independent of implantation site position along the uterine horn. Data expressed as mean ± SEM. ** *p* < 0.01, *** *p* < 0.001.

Since viral titers in the vaginal washes are a proxy assay for viral replication in the tissue, we next quantified viral DNA in vaginal tissue by HSV-2 qPCR. At gd7.5 (3dpi), HSV-2 DNA was detectable in vaginal tissues for all viral doses at comparable levels (Figure 3a). At gd12.5 (8dpi), HSV-2 DNA remained detectable within the vaginal tract for all viral doses, although it was present in lower quantities than at gd7.5, indicating the viral replication was past its peak in the tissue and had disseminated out of the vaginal tract (Figure 3b).

As HSV-2 DNA was detected in vaginal tissues of pregnant mice exposed to HSV-2, we decided to next examine the vaginal tract to assess local histopathology induced in response to the virus (Supplementary Figure S2). Vaginal tissues were excised at the time of sacrifice (3 or 8 dpi) and stained with H&E. Compared to vaginal tracts collected from uninfected gd7.5 (3 dpi) animals, infected vaginal tracts showed evidence of leukocyte infiltration even at the lower viral inoculation doses. Additionally, in contrast to the healthy, stratified vaginal epithelial barrier observed in uninfected animals, gd7.5 epithelium from infected mice appeared to be disintegrating, as fragmented epithelial cells were observed in the vaginal lumen (Supplementary Figure S2a). By gd12.5 (8dpi), leukocyte infiltration was prominent in the mucosa of infected animals. As well, the epithelial barrier was noticeably thinner than uninfected controls at gd12.5 and dose matched, infected samples at gd7.5, indicative of extensive epithelial damage as pregnancy progressed (Supplementary Figure S2b).

Since HSV-2 can infect placental tissues [37,38], we next asked whether the virus ascended from the vaginal tract to infect implantation sites at gd7.5 (3dpi), before establishment of the placenta at gd10.5 [36]. Healthy implantation sites were collected from animals sacrificed at gd7.5 and DNA was extracted from at least two individual implantation sites per mouse for qPCR quantification. At gd7.5 (3dpi), HSV-2 DNA was detected in implantation sites, indicting ascension from the vaginal tract (Figure 3c). Viral infection was observed in implantation sites in all groups, with comparable rate of infection among most viral doses (10^3^, 10^4^, 10^5^ pfu/mL). However, significantly higher quantities of viral DNA were detected in the implantation sites of animals infected with 10^2^ pfu/mL of HSV-2 (Figure 3c). Very little to no viral DNA was detected in implantation sites distal from the cervix, indicating that dissemination of the virus might be directional within the uterine horns. Indeed, correlation between implantation site position along the horn and viral load indicated that proximity to the cervix increased the risk of viral infection at gd7.5 (r^2^ = 0.1749; *p* = 0.0470) (Figure 3d).

Since we detected HSV-2 in gd7.5 implantation sites, we next asked if HSV-2 DNA persists in gd12.5 implantation sites after completion of placenta development. To quantify HSV-2 within the distinct compartments of the placenta, implantation sites were removed from pregnant animals and dissected to separate the maternal (decidua) and fetal (placenta) structures. By qPCR, HSV-2 was detected in gd12.5 decidua at all viral doses, but with higher expression at low viral doses than high (10^3^ vs. 10^5^ pfu/mL; *p* = 0.0055) (Figure 3e). As observed at gd7.5, several decidual samples from infected animals did not express any viral copies; however, unlike gd7.5, the position along the uterine horn did not correlate with viral load in decidual tissues (r^2^ = 0.0362; *p* = 0.5099) (Figure 3f), possibly indicating complete dissemination of the virus along the uterine horn in later stages of pregnancy. Within the fetal placenta of gd12.5 HSV-2-infected animals, HSV-2 DNA was detected with all viral doses (Figure 3g). Similar to our findings in the maternal decidua, viral load was significantly higher in low dose (10^3^ pfu/mL) placenta than high dose (10^5^ pfu/mL) (*p* = 0.0004), a number of samples did not express any viral copies (Figure 3g), and no correlation was observed between position along the uterine horn and viral load (r^2^ = 0.0093; *p* = 0.7688) (Figure 3h). Together, these results suggest that HSV-2 ascended from the vaginal tract into implantation sites 3dpi in a directional manner and persisted up to 8dpi in both maternal and fetal placental tissues.

### 3.4. Primary HSV-2 Infection in Early Pregnancy Affects Fetal Outcomes at gd12.5, but Not at gd 7.5

**Figure 4:**
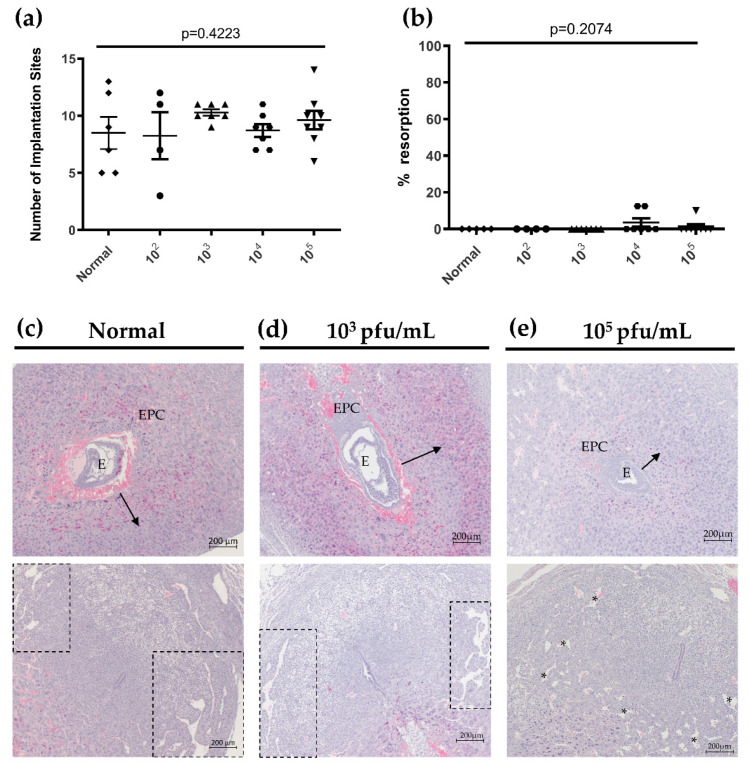
HSV-2 infection does not result in adverse pregnancy outcomes at gd7.5. (**a**) The number of healthy implantation sites in mice infected with 10^2^–10^5^ pfu/mL of HSV-2 did not vary significantly from uninfected normal controls at gd7.5 (n = 4–8 mice per group). (**b**) The percent resorption of pregnancies from infected mice did not differ from uninfected controls. Only mice infected with high viral doses (10^4^ and 10^5^ pfu/mL) had resorptions at gd7.5 (n = 4–8 mice per group). (**c**) Implantation sites from normal mice showed radial trophoblast invasion (arrow) and lateral decidual sinusoid formation (boxes). (**d**) In gd7.5 implantation sites from low dose (10^3^ pfu/mL) infected animals, radial trophoblast invasion (arrow) and sinusoid formation (boxes) were similar to controls. (**e**) In high dose (10^5^ pfu/mL) infected mice, embryos appeared smaller, trophoblast outgrowth was diminished (arrow), and lateral decidual sinusoids (*) were disorganized compared to control implantation sites. EPC: ectoplacental cone; E: embryo. Data expressed as mean ± SEM.

Since HSV-2 is known to affect pregnancy outcomes in women, particularly following primary infection during pregnancy, we next determined the effects of primary HSV-2 infection on successful pregnancy outcomes and placental and fetal development in our mouse model at both gd7.5 (3dpi) and 12.5 (8dpi). Uteri were dissected from gd7.5 pregnant animals and the number of healthy and resorbing implantation sites were enumerated as a measure of successful pregnancy. Resorptions were identified by their small and/or hemorrhagic appearance, indicating intrauterine fetal death. To account for variability in litter size amongst individual mice, resorptions are expressed as a percent of total implantation sites and averaged for each group (percent resorption). At gd7.5, there was no statistical difference in the number of healthy implantation sites (*p* = 0.4223 by one-way ANOVA) (Figure 4a) or in the percent resorption (*p* = 0.2074 by one-way ANOVA) (Figure 4b) between normal and HSV-2-infected mice with any viral dose. Importantly, at gd7.5, fetal resorptions were only observed in animals infected with high viral doses (10^4^/10^5^ pfu/mL); no resorptions were observed following inoculation with low viral doses (10^2^/10^3^ pfu/mL) (Figure 4b). This suggested that HSV-2 infection might result in different pregnancy outcomes when inoculation dose is low and viral infection spreads slowly (seen in low viral dose groups) or inoculation dose is high resulting in widespread infection (seen in high viral dose groups). As such, subsequent histology-based experiments were performed using mice infected with 10^3^ and 10^5^ pfu/mL of HSV-2 to simulate these different scenarios during pregnancy.

Histological analysis of healthy gd7.5 implantation sites in uninfected (normal), low dose infected (10^3^ pfu/mL) and high dose infected (10^5^ pfu/mL) animals was conducted using H&E-stained sections. Trophoblast invasion from the ectoplacental cone (EPC) into the maternal decidua is first observed at gd6.5 in mice and proper regulation of trophoblast invasion is essential for the development of a healthy placenta later in pregnancy [35]. In our model, at gd7.5, normal mice and mice infected with low dose HSV-2 (10^3^ pfu/mL) exhibited similar depth of radial trophoblast invasion into the maternal decidua and comparable development of lateral decidual sinusoids (Figure 4c,d). In contrast, although overt histopathology was not observed in the implantation sites of animals infected with high viral doses (10^5^ pfu/mL), there was diminished trophoblast outgrowth from the EPC (Figure 4e). Further, the embryos of highly infected mothers appeared smaller and lateral decidual sinusoids were disorganized in comparison to uninfected and low dose implantation sites (Figure 4e), indicating impairments in decidualization and angiogenesis in gd7.5 pregnant mice infected with high doses of HSV-2 [35,39].

**Figure 5:**
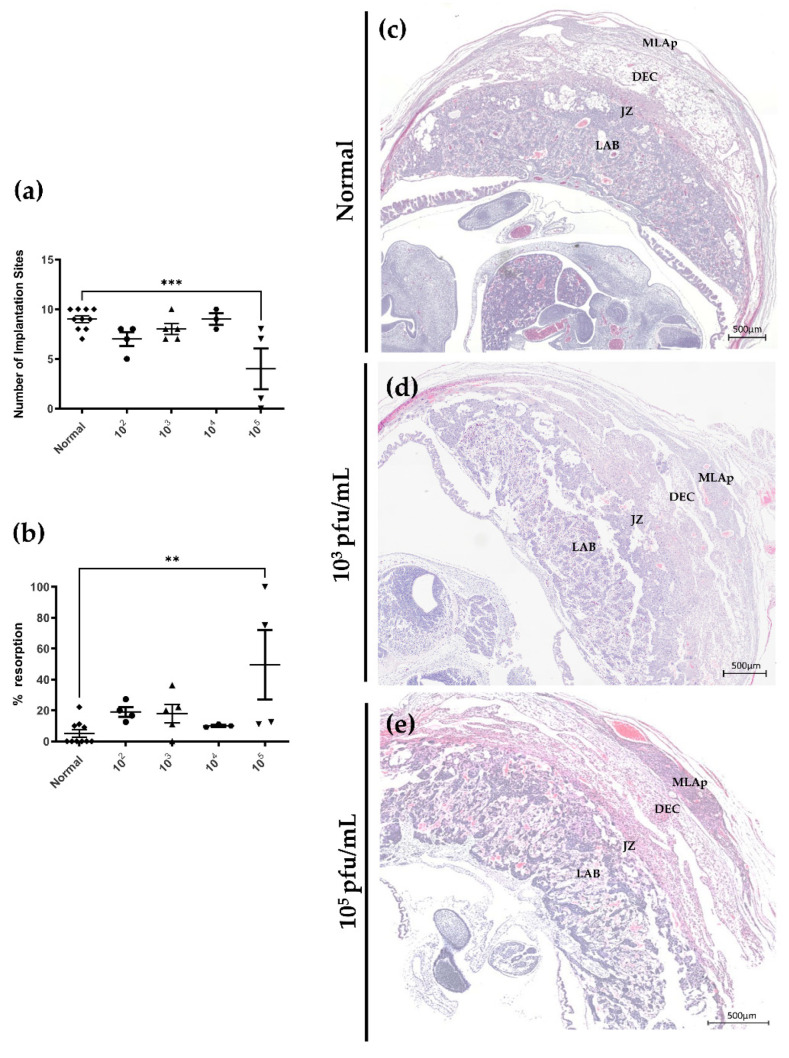
Pregnancy outcomes are affected by HSV-2 infection at gd12.5 resulting in placental pathology. (**a**) HSV-2 infection resulted in reductions in the number of healthy implantation sites compared to controls at gd12.5 (*n* = 3–10 mice per group). (**b**) Percent resorption was increased compared to controls in HSV-2-infected mice (*n* = 3–10 mice per group). (**c**) Implantation sites from normal gd12.5 mice consisted of 4 clearly defined and organized placental layers. (**d**) In mice infected with low dose HSV-2 (10^3^ pfu/mL), placental layers remained distinct but there was evidence of tissue dissociation. (**e**) In mice infected with high dose HSV-2 (10^5^ pfu/mL), placental layers were highly disorganized and tissue disintegration was evident. MLAp: mesometrial lymphoid aggregate of pregnancy; DEC: decidua; JZ: junctional zone; LAB: labyrinth. Data expressed as mean ± SEM. ** *p* < 0.01, *** *p* < 0.001.

In contrast to gd7.5 mice, at gd12.5 aberrant pregnancy outcomes became apparent in HSV-2-infected mice corresponding with increasing viral doses. Compared to normal animals, HSV-2-infected mice had decreased numbers of healthy implantation sites following viral inoculation, particularly in animals infected with a high viral dose. At a high viral inoculation dose of 10^5^ pfu/mL, the number of healthy implantation sites was significantly reduced compared to normal controls (*p* = 0.0008) (Figure 5a). Consistent with these findings, percent resorption increased in HSV-2-infected mice compared to controls in a dose-dependent fashion (Figure 5b). In 10^5^ pfu/mL-infected mice, percent resorption was significantly increased from normal controls (*p* = 0.0026) (Figure 5b). Unlike at gd7.5, at gd12.5, resorptions were not limited only to mice infected with high doses of HSV-2, indicating that 8 days of maternal infection with low dose HSV-2 was sufficient to cause fetal loss by gd12.5, and this was exacerbated in mice infected with high viral doses.

Based on the fetal loss observed in infected animals, we next examined the morphology of gd12.5 placental tissues. In gd12.5 implantation sites collected from normal mice, maternal and fetal placental layers appear as organized, distinct tissue regions (Figure 5c). The maternal layers of the placenta consist of the mesometrial lymphoid aggregate of pregnancy (MLAp), which forms between two layers of the myometrium, and the decidua, which forms the bulk of the maternal placenta. Conceptus-derived layers include the junctional zone (JZ), comprised of trophoblast giant cells (TGCs) and spongiotrophoblast, and the labyrinth, the site of maternal–fetal exchange [39,40]. In 10^3^ pfu/mL HSV-2-infected mice, placental layers remained distinctive, but tissue dissociation, particularly within conceptus-derived layers (JZ and labyrinth), appeared in numerous sections (Figure 5d). In 10^5^ pfu/mL HSV-2-infected mice, placental layers were disorganized and indistinct as tissue disintegration, hemorrhage, and edema were evident across the whole implantation site (Figure 5e). Thus, while HSV-2 infection at gd7.5 was associated with minimal fetal loss and little histopathology, both controlled (low dose) and widespread (high dose) infection resulted in fetal loss and gross placental pathology at gd12.5.

### 3.5. HSV-2 Infection of Maternal and Fetal Placental Tissues Correlates with Histopathology Consistent with Loss of Integrity and Decreased Fetal Growth at gd12.5

**Figure 6:**
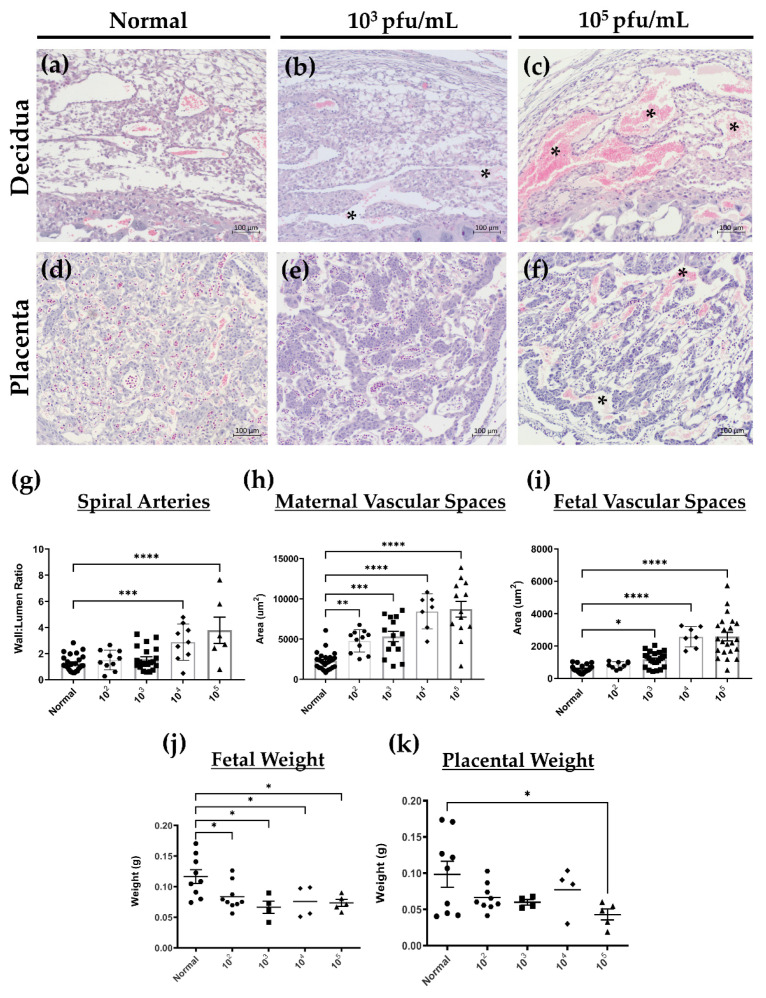
HSV-2 infection compromises tissue integrity and vascular remodeling resulting in decreased fetal growth at gd12.5. (**a**) H&E staining of decidua from normal mice at gd12.5. (**b**) Decidua from mice infected with low dose HSV-2 (10^3^ pfu/mL) showed signs of hemorrhage and necrosis (*). (**c**) With high dose HSV-2 (10^5^ pfu/mL) tissue pathology became more apparent with rampant hemorrhage and necrosis in decidual tissue (*). (**d**) H&E staining of the placenta labyrinth in normal mice consists of intricate vascular branching patterns and thin interhemal membranes. (**e**) In animals infected with low dose HSV-2 (10^3^ pfu/mL), the labyrinth consisted of long, straight vascular spaces with less vessel branching and thicker interhemal membranes than controls. (**f**) In the labyrinth of high dose (10^5^ pfu/mL) infected mice, there were regions of extensive hemorrhage (*) and vessel branching was impaired. (**g**) Compared to controls, high dose (10^4^ and 10^5^ pfu/mL), but not low dose (10^2^ and 10^3^ pfu/mL), infection with HSV-2 significantly increased wall-lumen ratios of spiral arteries compared. (**h**) Maternal vascular spaces in the labyrinth were increased significantly from controls with all doses of HSV-2. (**i**) Fetal vascular spaces in the labyrinth were increased significantly for all viral doses greater than 10^2^ pfu/mL compared to normal controls. (**j**) Gd12.5 fetuses dissected from implantation sites of infected animals weighed less than control fetuses at all viral doses (*n* = 4–9 fetuses per group). (**k**) Fetal placentas dissected from implantation sites of animals infected with a high (10^5^ pfu/mL) viral dose had significantly reduced weights compared to controls (*n* = 4–9 placentas per group). Data expressed as mean ± SEM. * *p* < 0.05, ** *p* < 0.01, *** *p* < 0.001, **** *p* < 0.0001.

Our data on negative pregnancy outcomes and gross abnormalities in placental structures at gd12.5 led us to further investigate the maternal decidua and fetal placental layers in control and infected animals at gd12.5. Compared to normal controls (Figure 6a), H&E staining of maternal decidua revealed areas of hemorrhage and necrosis in pregnant mice infected with 10^3^ pfu/mL of HSV-2 (Figure 6b), which were extensive in mice infected with 10^5^ pfu/mL HSV-2 (Figure 6c), thus indicating increasing loss of maternal tissue integrity proportionate to HSV-2 inoculation dose. Similarly, H&E staining of the fetal placenta in normal animals showed a placental labyrinth structure comprised of tightly packed branching structures with thin interhemal membranes separating maternal and fetal vasculature (Figure 6d). In low dose (10^3^ pfu/mL) infected animals, vascular branching was diminished as evidenced by large, straight vascular spaces, and interhemal membranes separating maternally were thicker than in controls (Figure 6e). Labyrinth pathology and disorganization were more apparent at high infectious doses (10^5^ pfu/mL) where necrosis and hemorrhage in maternal vasculature were extensive (Figure 6f), leading to impaired branching structures. There was also evidence of leukocyte infiltration not observed in controls or in animals infected with low viral dose.

To quantitatively assess measures of tissue integrity, which could lead to impairments in functional capacity, we examined spiral artery remodeling within the decidua. Spiral arteries represent the terminal branches of the maternal uterine arteries and are typically remodeled by mid-pregnancy into low pulsatile, high volume vessels able to accommodate increased blood flow to the developing fetus [36]. In normal animals at gd12.5, spiral arteries lose their smooth muscle wall and luminal diameters are increased. This modification is assessed by a wall-lumen ratio calculated using the total vessel and luminal areas as described in the Materials and Methods [34]. In comparison to normal control animals, which had an average wall-lumen ratio of 1.25, animals infected with HSV-2 had wall-lumen ratios that increased with increasing viral dose. With high viral dose, this change became significant as vessel lumens were greatly diminished and arterial walls remained thicker than controls. Compared to normal controls, animals infected with 10^4^ and 10^5^ pfu/mL HSV-2 had an increased average wall-lumen ratio of 2.88 (*p* = 0.0008) and 3.79 (p < 0.0001), respectively (Figure 6g). Together, these results indicate that higher inoculation doses leading to widespread HSV-2 infection, affect the integrity of the maternal placental structures and will likely disturb blood flow to the developing fetus.

To corroborate whether the histological impairments observed in branching patterns of the fetal placenta labyrinth were affected by HSV-2 infection, maternal and fetal vascular spaces were quantified, as described in the Materials and Methods section [41]. Compared to maternal vascular spaces in normal animals (2309.97 µm^2^), there were significant increases in maternal vascular spaces in the labyrinth with all viral doses (Figure 6h). Fetal vascular spaces were also increased compared to controls in animals infected with all viral doses except 10^2^ pfu/mL (*p* = 0.8304) (Figure 6i). This confirms that vascular branching was reduced with HSV-2 infection, especially at high viral doses, likely resulting in diminished diffusion capabilities of the fetal placenta.

Since we observed histopathology that indicated deficiencies in maternal blood flow and exchange capacity in infected animals, and because HSV-2 infection in human pregnancies is linked to several adverse outcomes including IUGR and low birth weight [14,16], we next investigated the effect of primary HSV-2 infection on fetal weight and development in our animal model. At the time of sacrifice, placental tissues and fetuses were dissected and weighed separately. Compared to normal controls, there was a significant decrease in fetal weight in animals infected with HSV-2 at all viral doses (Figure 6j). In animals infected with 10^5^ pfu/mL HSV-2, there was also a significant 2.3-fold reduction in placental weight compared to controls (*p* = 0.0278) (Figure 6k). Together, these results indicate that HSV-2-induced damage to placental blood flow and vascular structures resulted in decreased fetal growth and placental size, especially with high viral dose.

### 3.6. High Dose HSV-2 Infection Is Associated with Increased Levels of Pro-Inflammatory Cytokines in Decidual and Placental Tissues on gd12.5

**Figure 7:**
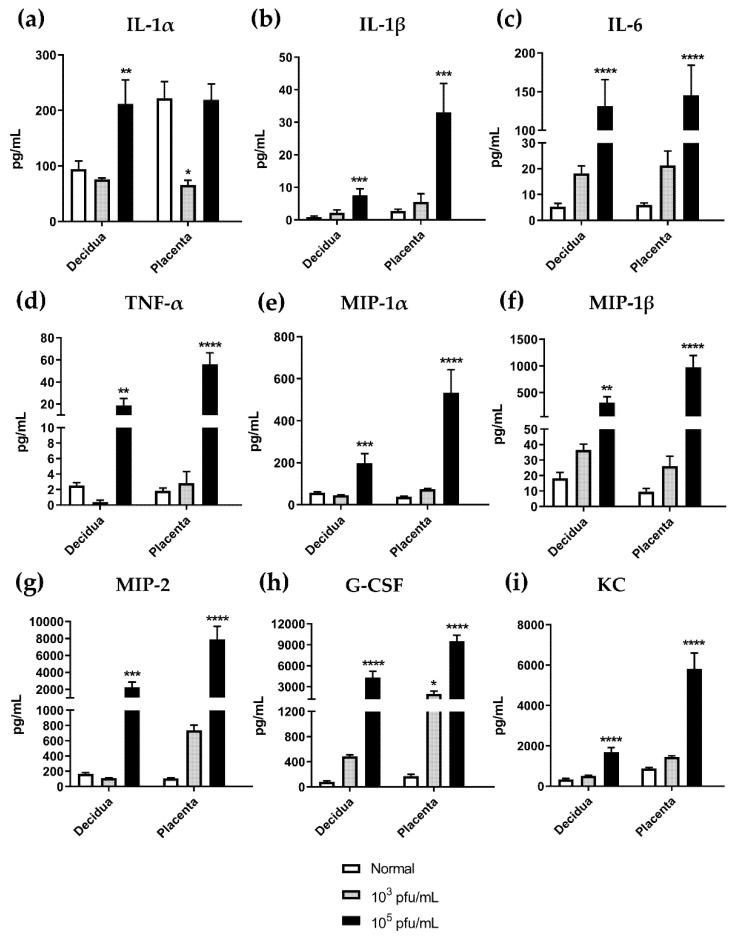
HSV-2 infection alters cytokine/chemokine profiles in the decidua and placenta at gd12.5. Cytokines in tissue homogenates from normal mice and mice infected with low (10^3^ pfu/mL) and high (10^5^ pfu/mL) dose HSV-2 were analyzed by 32-plex array and 9 analytes were selected for further analysis: (**a**) IL-1α, (**b**) IL-1β, (**c**) IL-6, (**d**) TNF-α, (**e**) MIP-1α, (**f**) MIP-1β, (**g**) MIP-2, (**h**) G-CSF, and (**i**) KC. (**a**) IL-1α was significantly increased in the decidua of high dose infected animals and significantly decreased in the placenta of low dose infected animals. (**b**–**i**) Compared to normal controls, high dose HSV-2 infection significantly increased cytokine/chemokine levels in decidua and placenta homogenates. (**h**) Compared to controls, G-CSF was significantly increased in placenta tissue homogenates with both low and high dose HSV-2 infection. Data expressed as mean ± SEM. * *p* < 0.05, ** *p* < 0.01, *** *p* < 0.001, **** *p* < 0.0001.

Our finding of gross anomalies and evidence of leukocyte infiltration in placental tissues at gd12.5 next led us to postulate whether these changes would be associated with the production of inflammatory mediators. We evaluated cytokine/chemokine profiles in maternal decidua and fetal placenta tissue homogenates collected from gd12.5 normal, uninfected mice and mice infected with HSV-2 at representative low (10^3^ pfu/mL) and high (10^5^ pfu/mL) viral doses. Raw data generated from a 32-plex cytokine/chemokine array were analyzed through generation of heatmaps for each tissue (data not shown). Based on observed trends in clustering and expression patterns between the infected groups, nine analytes were selected for further analysis: IL-1α, IL1-β, IL-6, TNF-α, MIP-1α, MIP-1β, MIP-2, G-CSF, and KC (CXCL1) (Figure 7). Importantly, these analytes have roles related to the recruitment of polymorphonuclear cells and promotion of inflammatory responses. Overall, cytokine levels were comparable between decidua and placental tissues. With the exception of IL-1α (Figure 7a), all cytokines/chemokines in decidua and placenta tissues had similar patterns of dose-dependent increases in expression. IL-1α levels in the decidua of normal and low dose (10^3^ pfu/mL) HSV-2-infected animals were comparable (*p* = 0.9208), but expression was significantly increased with high dose (10^5^ pfu/mL) HSV-2 infection (*p* = 0.0090). Interestingly, in the placenta, low dose HSV-2 infection appeared to decrease expression of IL-1α compared to controls (*p* = 0.0108), while high dose had no significant effect on IL-1α levels (*p* = 0.9961). With all other cytokines/chemokines analyzed, infection with low dose HSV-2 (10^3^ pfu/mL) appeared to increase expression compared to normal, uninfected controls (Figure 7b–i); however, significant increases were only observed for G-CSF expression in the placenta of animals infected with low dose HSV-2 infection (*p* = 0.0439) (Figure 7h). Following high dose HSV-2 infection, all cytokines/chemokines were significantly increased from normal controls in all tissues tested (Figure 7b–i). Thus, taken together, low dose HSV-2 appears to initiate a low-grade inflammation in maternal and fetal placental tissues, while high dose HSV-2 infection significantly intensifies the expression of pro-inflammatory cytokines, corresponding to enhanced placental damage observed in histological analysis.

### 3.7. In Utero HSV-2 Transmission into Fetuses Is Observed at gd12.5 and Is Accompanied by Inflammatory Responses

**Figure 8:**
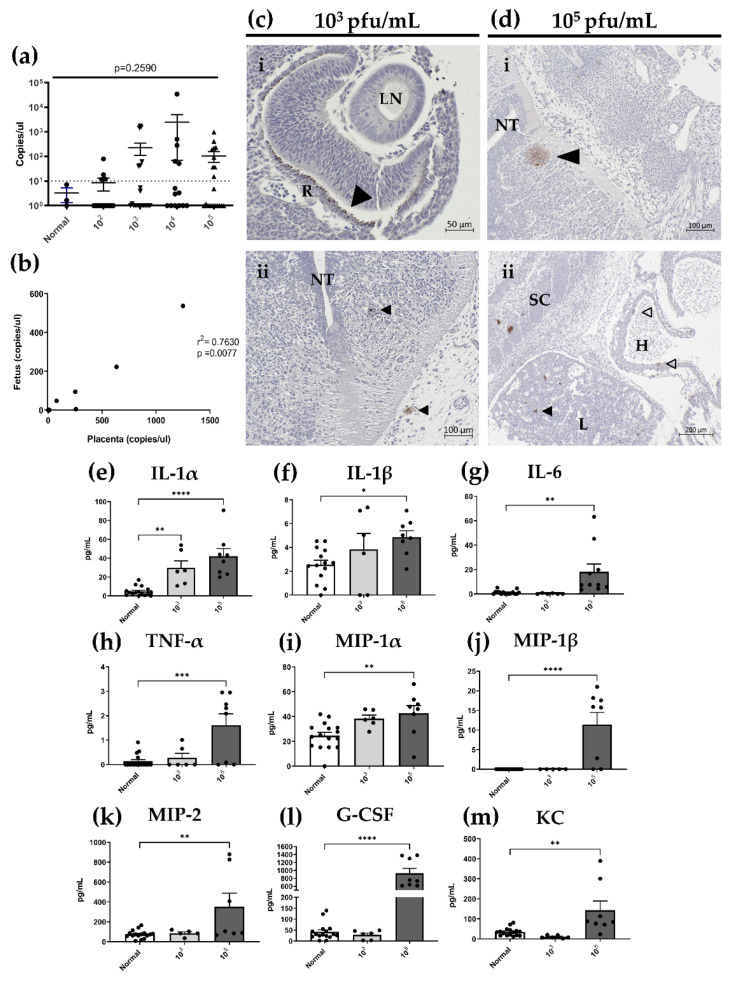
In utero HSV-2 transmission into fetuses results in congenital HSV-2 infection and inflammatory responses at gd12.5. (**a**) HSV-2 DNA was detected in fetal homogenates by qPCR with comparable levels for all viral doses. (**b**) There was a significant correlation between HSV-2 DNA levels in matched fetal and placental tissues, but HSV-2 DNA was not detected in fetal homogenates in the absence of placental infection. Detection of HSV-2 DNA in placental tissues did not guarantee HSV-2 detection in matched fetal homogenates. (**c**) In fetuses from mice infected with low dose HSV-2 (10^3^ pfu/mL), HSV-2 was detected in (i) the developing retina and (ii) neuroepithelium surrounding the neural tube (arrowheads). (**d**) HSV-2 staining in fetuses from mice infected with high dose HSV-2 (10^5^ pfu/mL) showed disseminated expression of HSV-2 in fetal tissues including the (i) neuroepithelium around the neural tube (arrowhead), (ii) heart (open arrowheads), and liver (closed arrowhead). (**e**) IL1α expression levels were significantly increased compared to controls in fetuses from mice infected with both low (10^3^ pfu/mL) and high (10^5^ pfu/mL) dose HSV-2. (**f**–**m**) Expression of all other pro-inflammatory cytokines and chemokines in fetal homogenates was significantly increased in fetuses from pregnancies complicated by high, but not low, dose HSV-2 infection. H: heart, L: liver, LN: lens, NT: neural tube, R: retina, SC: spinal column. Data expressed as mean ± SEM. * *p* < 0.05, ** *p* < 0.01, *** *p* < 0.001, **** *p* < 0.0001.

The observation that placental structures are compromised and fetal weights decreased in HSV-2-infected animals led us to ask whether there was in utero transmission of HSV-2 to the fetus across the placental barrier. Indeed, after dissection of the fetus from implantation sites at gd12.5, HSV-2 DNA was detected in fetuses with comparable viral loads for all viral doses (Figure 8a), indicating that vertical transmission of HSV-2 occurs even at low viral doses. The position along the uterine horn did not appear to be a factor affecting transplacental viral transmission as transmission occurred both proximally and distally to the cervix. There was, however, significant correlation between HSV-2 copy numbers for matched placenta and fetal samples from infected animals (*p* = 0.0077) (Figure 8b). Importantly, HSV-2 DNA was not detected in fetuses in the absence of placental infection and infection in the placenta did not always result in fetal acquisition of HSV-2.

Further examination of HSV-2 infection of fetal tissues by HSV-2 IHC showed that with low viral dose (10^3^ pfu/mL), HSV-2 was not fully disseminated within fetal tissues. Clear staining patterns were observed in the eye and confined exclusively to the retina rather than the lens (Figure 8ci). In some instances, HSV-2 was also detected in neuronal tissues of the CNS, both in developing brain structures and in the neuroepithelium surrounding the neural tube (Figure 8cii). With high viral dose (10^5^ pfu/mL), HSV-2 was fully disseminated within fetal tissues, infecting the eye, CNS, liver and heart (Figure 8di,ii). Thus, maternal infection with low and high doses of HSV-2 results in transplacental transmission of HSV-2 and congenital herpes infection in our mouse model.

Since HSV-2 was detected in fetuses by qPCR and IHC, we next investigated whether viral infection in fetal tissues stimulated the production of inflammatory cytokines and chemokines using the same analytes detected in decidual and placental tissues: IL-1α, IL1-β, IL-6, TNF-α, MIP-1α, MIP-1β, MIP-2, G-CSF, and KC (CXCL1). Overall, cytokine levels in fetal homogenates were lower than those detected in the decidua and placenta; however, as in the decidua and placenta, inflammatory mediators in fetal tissues increased in a dose-dependent fashion. For all analytes measured, there were significant increases in cytokine/chemokine expression in fetuses from mice infected with high dose HSV-2 (10^5^ pfu/mL) compared to controls (Figure 8e–m). Of the nine analytes, only IL-1α expression increased in comparison to controls in fetuses from animals infected with low dose HSV-2 (10^3^ pfu/mL) (Figure 8e; *p* = 0.0023). Thus, taken together, in utero HSV-2 transmission to fetuses in animals infected with high doses of HSV-2 resulted in significantly increased expression of pro-inflammatory cytokines within fetal tissues.

## 4. Discussion

In this study, we describe a novel, clinically relevant mouse model demonstrating that pregnant mice are 100-fold more susceptible to HSV-2 primary infection compared to nonpregnant mice in the diestrus stage of the reproductive cycle. As early as 3 dpi, HSV-2 was able to ascend the vaginal tract and establish infection in developing implantation sites. By 8dpi, HSV-2 infection was observed in both the maternal decidua and fetal placenta, leading to widespread pathology, significantly decreased fetal weights, and increased expression of inflammatory cytokine/chemokine profiles in implantation sites. Further, HSV-2 DNA was present in the developing fetus, even in experimental groups inoculated with low viral doses. This is the first study to demonstrate dissemination of HSV-2 into the uterus during pregnancy and provide evidence of productive placental and fetal infection resulting in pro-inflammatory cytokine responses in both the maternal and fetal tissue, in an in vivo model. As a result, this model will provide a very useful tool to understand the mechanisms by which primary HSV-2 infection in women contributes to numerous adverse pregnancy outcomes including IUGR, spontaneous abortion, and congenital HSV-2 infection of the neonate.

It is well established that the female sex hormones, estradiol and progesterone, play key roles in regulating susceptibility of the FRT to sexually transmitted infections, including HSV-2 [42,43,44]. Experimental studies in mice have shown that during the high-progesterone phases of the estrous cycle (diestrus) or following treatment with exogenous progesterone, mice are more susceptible to intravaginal HSV-2 infection [2,3]. Indeed, during pregnancy, which is marked by heightened levels of endogenous progesterone, women are more susceptible to infectious agents, including microbial products and viruses, and the severity of infections during pregnancy is intensified [7,45,46,47]. To date, very few studies have examined HSV-2 infection and its effect on pregnancy outcomes using mouse pregnancy models. In two previous studies conducted in pregnant mice inoculated at varying gestation days, it was demonstrated that pregnancy enhanced HSV-2 susceptibility at rates greater than progesterone treatment in nonpregnant animals [48,49]. However, these studies relied solely on survival outcomes as a measure of susceptibility and did not investigate the effect of inoculation dose on the outcomes of pregnancy. In one additional study, it was shown that pregnant mice inoculated with a high dose of HSV-2 (5 × 10^5^ pfu/mL) at two timepoints during early and mid-pregnancy had high rates of maternal mortality and fetal resorption [27]. However, this study used a high viral dose previously shown to be lethal in nonpregnant, diestrus-staged mice [2]. Moreover, no follow up experiments were performed to further investigate the observed fetal loss. Our experiments validated and expanded on these previous studies by demonstrating increased susceptibility in pregnant mice compared to nonpregnant, diestrus-staged controls using a variety of metrics including survival, pathology scoring, and viral titers in vaginal washes. Unlike mice treated with exogenous hormones, diestrus-staged animals represent a more physiologically relevant level of progesterone exposure. Further, we examined in detail the effect of inoculation dose on pregnancy outcomes, including fetal loss, observed in our model. While the effects of lower inoculation doses were marginal at earlier timepoints during pregnancy (gd7.5), overall adverse outcomes at gd12.5, including histopathology in the placenta, diminished branching in the placenta labyrinth, decreased fetal weight, and transmission of HSV-2 across the placenta, were apparent across all inoculation ranges, even the lowest doses. Thus, even low-dose HSV-2 exposure during pregnancy could lead to adverse outcomes through several pathways.

One of the interesting findings in our model was the presence of viral DNA in implantation sites as early as 3 dpi (gd7.5). Differential infection rates in implantation sites in accordance with distance from the cervix confirm that HSV-2 is able to ascend from the vaginal tract, even with low viral dose, and subsequently progress along the uterine horn. Few studies have investigated HSV-2 infection of early first trimester placental tissues in humans; however, examination of tissues obtained from HSV-2-positive women undergoing elective termination or experiencing first trimester pregnancy loss (between 6 and 12 weeks gestation) revealed that HSV-2 can be detected in early pregnancy tissues by nested PCR. Further examination using IHC and in situ hybridization showed that viral DNA localizes to the nuclei of decidual and cytotrophoblast cells invading maternal tissues [37,50]. In line with this finding, our mouse model demonstrated that HSV-2 is capable of infecting implantation sites, including maternal decidua and developing placental tissues, at an early timepoint in pregnancy. A recent study by Lin et al. also demonstrated that cytomegalovirus infection of human extravillous trophoblast in culture inhibited their proliferation and migration [51]. Therefore, it is possible that direct HSV-2 infection of trophoblast cells within implantation sites accounts for the aberrant trophoblast invasion seen at gd7.5 (Figure 4). Further to this point, careful coordination of trophoblast differentiation and invasion in early pregnancy is crucial for formation of the various conceptus-derived placental structures, including the labyrinth and spongiotrophoblast [39,40]. Consequently, it is likely that HSV-2 infection of implantation sites at gd7.5 affects proper placentation and function later in gestation.

In support of this, at gd12.5 in our model, we observed highly disorganized and fragmented placental structures in animals infected with low and high dose HSV-2 (Figure 5). This was particularly evident in the labyrinth where diminished branching resulted in significantly increased maternal and fetal vascular spaces. Within the labyrinth, efficient maternal–fetal exchange occurs at capillary interfaces, not within larger vessels, thus HSV-2 infection appears to limit the surface area available for nutrient exchange [36]. Indeed, with all viral doses, we observed significant reductions in fetal weight and evidence of IUGR, corresponding with the observed altered or impaired development of placental labyrinth structures (Figure 6). This is in agreement with other mouse studies demonstrating that deficiencies of villous structures, including diminished exchange capacity, altered vascular perfusion, and increased vascular spaces [52,53], result in IUGR. Importantly, IUGR is one of the most consistently reported clinical complications of HSV-2 infection [14,18,19]. Thus, our mouse model provides an important tool to better understand the mechanisms leading to placental dysfunction and IUGR in the context of HSV-2 infection.

In severe cases of placental dysfunction [54,55] and in pregnancies complicated by HSV-2 infection, particularly primary infection, spontaneous fetal loss or stillbirth may occur [16,17,56]. This was shown in our model in the context of high-dose HSV-2 exposure where we observed a significant increase in the proportion of fetal resorptions at gd12.5 (Figure 5). This adverse outcome differs from low-dose HSV-2 exposure, which resulted in IUGR in the absence of significant fetal loss, as discussed above. Thus, while impairments in placental structures may partially explain the IUGR and fetal loss observed in our model, it is also important to note that HSV-2 persisted in placental tissues at gd12.5 and affected both the maternal and fetal placental components. This is in line with studies conducted in term human pregnancies complicated by maternal HSV-2 infection, where expression of HSV-2 is demonstrated by IHC and PCR in both the maternal and fetal sides of the placenta [23,57,58,59]. In these studies, placental and fetal infection occurred even in cases of asymptomatic maternal HSV-2 infection [23], and detection of HSV-2 DNA in the fetal placenta was linked to cases of intrauterine deaths, including spontaneous abortion and stillbirths [38,56,58,59]. Further, the expression of viral antigens coincided with placentitis, or an inflammation of the placenta, and placental insufficiency due to disordered fetoplacental blood circulation and decreased numbers of fetal vessels [38,58,59]. This parallels what we observe in our model at gd12.5, where the placental labyrinth structure is impaired and inflammation is evident throughout the maternal and fetal placental compartments (Figure 6). Indeed, unlike gd7.5 where HSV-2 infection of implantation sites resulted in relatively little histopathology, by gd12.5, we saw gross histological pathologies including leukocyte infiltration, hemorrhage, edema, and necrosis in the placenta. Importantly, these same findings, particularly cell necrosis, have been reported in the placenta of a neonate diagnosed with intrauterine HSV infection [60]. Thus, our model appears to recapitulate the clinical effects of HSV-2 infection on placental tissues.

The histopathology we observed in our model is likely the result of tissue breakdown due to inflammation. Indeed, corresponding with the observed histopathology at gd12.5, the results from our cytokine/chemokine array show consistent increases in the production of key neutrophil chemoattractants (KC (CXCL1), MIP-2 (CXCL2), MIP-1α (CCL3), and MIP-1β (CCL4)), and pro-inflammatory cytokines (IL-1α, IL-1β, IL-6, TNF-α) in both maternal and fetal tissues. Importantly, with low-dose (10^3^ pfu/mL) HSV-2 infection, this inflammatory immune response, although enhanced by infection, was not significantly different from controls. In contrast, high-dose (10^5^ pfu/mL) infection led to significant upregulation of pro-inflammatory cytokines. This suggests that with low-dose viral infection, there is a sustained yet controlled inflammatory response that is significantly exacerbated with higher viral dose, thus accounting for the observed differences in fetal outcomes. Indeed, multiple studies have suggested that diverse combinations and expression levels of pro-inflammatory cytokines can play a key role in differential outcomes of pregnancy. For instance, in the plasma of mothers with preeclampsia, concentrations of IL-6, but not TNFα or IL-1β, were higher than those reported in normal patients [61]. Alternately, induction of preterm birth by mid-pregnancy administration of heat-killed *E. coli* resulted in significantly increased levels of IL-1α, IL-1β, and IL-6 in the uterus, while TNF-α levels were similar to controls [62]. Additionally, low-grade inflammation in the placenta, as observed with low doses of HSV-2 in our study, has been observed in pregnancies complicated by maternal obesity that result in adverse pregnancy outcomes, including congenital abnormalities and fetal macrosomia, in the absence of more severe outcomes [63,64]. Further, in a study conducted using first trimester placental tissues obtained from normal pregnancies and spontaneous abortions, only placental tissues from spontaneous abortions were associated with unstimulated production of inflammatory mediators [65]. This may point to a role of other placental factors in enhancing pro-inflammatory cytokine production, which underlies severe placental pathology and fetal death, as observed following high-dose infection in our study.

It is important to consider that cytokines do not act as single factors, but rather act as a network through multiple, redundant pathways; thus, it is important to examine the entire cytokine profile, rather than focus on the expression of a single mediator. However, the alteration in IL-1α levels in the placenta of normal control animals is an interesting finding of this study that merits discussion (Figure 7). The IL-1 system encompasses many important roles in mammalian reproduction, chiefly in uterine receptivity and embryo implantation [66]. While the role of IL-1β is fairly well defined, the participation of IL-1α in the promotion of normal pregnancy is less studied. IL-1α can be detected in first trimester and term human placentas with expression observed in trophoblast and stromal cells by histological staining [67,68]. High levels of IL-1α have also been detected in healthy mouse placenta from gd12.5 and 17.5 [69]. It is theorized that the presence of the IL-1 system, including IL-1α, at the maternal–fetal interface might suggest multiple roles for this cytokine in both eliciting immune responses to protect against pathogens, and stimulating placenta development and trophoblast proliferation [68,70]; however, the expression and particular role of IL-1α in pregnancies complicated by HSV-2 infection warrants much further experimentation.

There is much interest in the connection between vasculopathology and inflammation in the context of the fetoplacental unit [71,72]. It is known that inflammation can directly affect placental hemodynamics and spiral artery remodeling, resulting in IUGR and fetal loss [72,73,74]. In line with this, in dams given a high viral dose, we observed impaired spiral artery remodeling, as indicated by an increased wall to lumen ratio, and extensive hemorrhage in both the maternal and fetal placental compartments (Figure 6). Importantly, these alterations were minimized in our low-dose animals where infection and inflammation were controlled, resulting in less severe areas of hemorrhage and nonsignificant differences in spiral artery remodeling. Therefore, our model suggests that HSV-2 infection of implantation sites at gd7.5 likely impairs trophoblast proliferation and invasion, ultimately leading to compromised placental development and abnormal structures at gd12.5. This alteration affects maternal–fetal circulation, resulting in IUGR and decreased fetal weight even with low viral doses. When dams are given a high viral dose, viral accumulation in select implantation sites further enhances inflammation, tissue breakdown, and deterioration of placental structures, which ultimately leads to fetal loss not observed with lower viral doses.

One of the most striking findings in our model of HSV-2 infection is that the virus is transmitted across the placenta to infect the developing fetus, even at low viral doses when the placenta is intact. Interestingly, however, is that the severity of fetal HSV-2 infection appears to be dependent on viral dose. With low-dose HSV-2, fetal infection was limited to the eye and CNS, while high-dose HSV-2 infection resulted in enhanced viremia, further affecting the liver and heart (Figure 8). This is reminiscent of clinical findings observed in human pregnancies. Localized infection of the skin, eyes or mouth, with or without CNS involvement, as observed following our low-dose, controlled infection, is rarely fatal in neonates but may have associated neurological morbidities [26]. Multi-organ, disseminated disease, as observed in our high dose pregnancies, is the most serious form of neonatal herpes infection accounting for around 25% of cases with an associated mortality rate of 35% [9,13,24]. How HSV-2, or other viral pathogens, are transmitted to the fetus in utero is relatively unknown. Studies investigating infection with Zika virus showed that inflammation in placental compartments can cause trophoblast apoptosis and autophagy, which impairs the placental barrier, resulting in in utero viral transmission [75]. This is likely what is happening in our model, as inflammatory cytokines are elevated in placental tissues, and there is evidence of trophoblast cell death due to HSV-2 infection in our histological specimens; however, further studies need to identify the exact underlying mechanisms of transmission. Since the risk of morbidity and mortality in neonatal HSV-2 infection is highest when HSV-2 is acquired in utero [9,11,12,14,21], our model, which demonstrates vertical transmission of HSV-2, even with low viral doses, provides a valuable tool to understand the exact mechanisms contributing to in utero HSV-2 acquisition.

In conclusion, we demonstrate for the first time in an in vivo mouse model that HSV-2 infection in the mother, both at low and high viral doses, results in viral infection of maternally and fetally derived placental tissues and in utero transmission of HSV-2 from the mother to the fetus. Low-dose viral infection results in low-grade, controlled inflammation and IUGR, while rampant, high-dose HSV-2 infection is associated with extensive inflammation and more severe fetal outcomes, including fetal loss, at mid-pregnancy. It is our hope that this model can be used to study mechanisms of viral transmission and assist in the development of a vaccine for the prevention of severe fetal morbidity and mortalities associated with intrauterine HSV-2 infection.

## Figures and Tables

**Table 1:** Cumulative pathology scores for nonpregnant, diestrus-staged and pregnant mice ^a^.

Group	Pathology Score	No. of Mice	No. of Days	Cumulative Pathology	Avg. Pathology/Mouse (avg. ± SEM)
*Diestrus*					
10^2^ pfu/mL	0	6	8	0	0.7 ± 0.62
	5	1	1	5	
10^3^ pfu/mL	0	5	8	0	0.9 ± 0.59
	1	1	2	2	
	2	1	2	4	
10^4^ pfu/mL	0	3	8	0	1.5 ± 0.72
	1	1	3	3	
	2	1	1	2	
	4	1	1	4	
10^5^ pfu/mL	0	1	8	0	5.0 ± 0.98
	2	1	3	6	
	3	1	2	6	
	4	2	1	8	
	5	2	1	10	
	5	1	2	10	
*Pregnant*					
10^2^ pfu/mL	1	1	2	2	2.8 ± 0.48 ^b^
	2	1	1	2	
	2	1	2	4	
	3	1	1	3	
10^3^ pfu/mL	1	1	2	2	4.2 ± 1.0 ^b^
	3	1	1	3	
	4	2	1	8	
	4	1	2	8	
10^4^ pfu/mL	4	1	1	4	6.7 ± 1.3^b^
	4	2	2	16	
10^5^ pfu/mL	4	1	1	4	8.2 ± 1.7
	4	1	3	12	
	5	4	1	20	
	5	2	3	30	

^a^ In order to compare groups, cumulative scores of pathology were determined by tabulating the number of mice with the highest pathology score they achieved and the number of days that score was observed. Mice that did not survive to the end of the challenge were given the highest pathology score at the time of death and assigned that score for each day that remained in the duration of the experiment. In this way, overall pathology was accurately reported for each group. The sum of all the scores for all the mice in each group was the total level of pathology for that group and then, the average pathology score per mouse for each group was calculated by dividing total pathology by the number of mice in each group. ^b^ *p* < 0.05 compared to nonpregnant, diestrus-staged controls.

## Data Availability

Not applicable.

## Supplementary Materials

The following are available online at https://www.mdpi.com/article/10.3390/v13101929/s1, Supplementary Figure S1: Genital pathology scores for nonpregnant, diestrus-staged and pregnant mice infected with varying doses of HSV-2, Supplementary Figure S2: Pathology is evident in the vaginal tract of HSV-2-infected pregnant mice by histology.
