# Supplementary figures and images for: Primary HSV-2 Infection in Early Pregnancy Results in Transplacental Viral Transmission and Dose-Dependent Adverse Pregnancy Outcomes in a Novel Mouse Model
Source: Viruses. 2021 Sep 25;13(10):1929. doi: 10.3390/v13101929 (PMC8538385; doi:10.3390/v13101929)

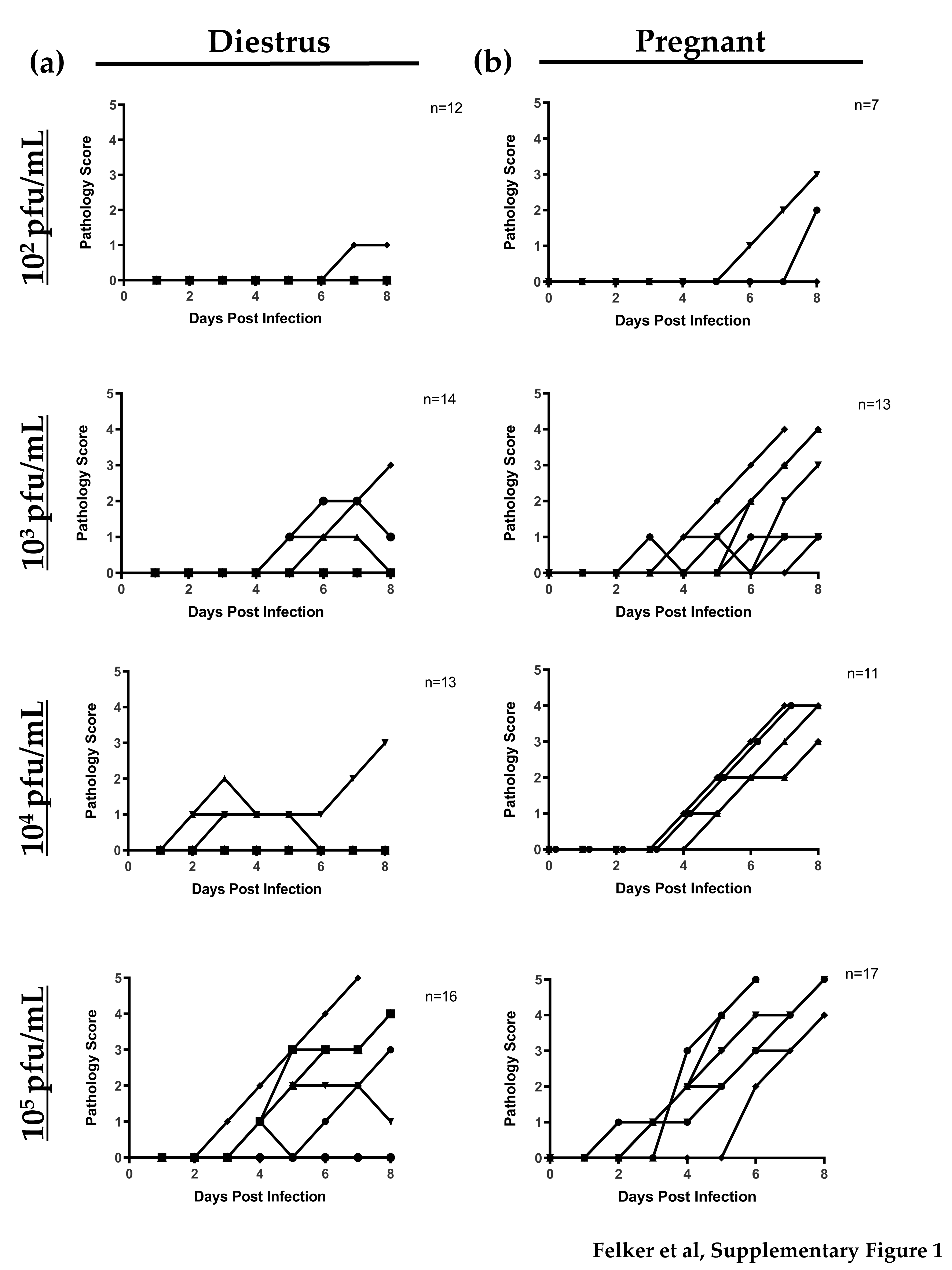

Supplement: Supplementary file 1 [file viruses-13-01929-s001.zip › Felker et al Supp Figure 1.tif]

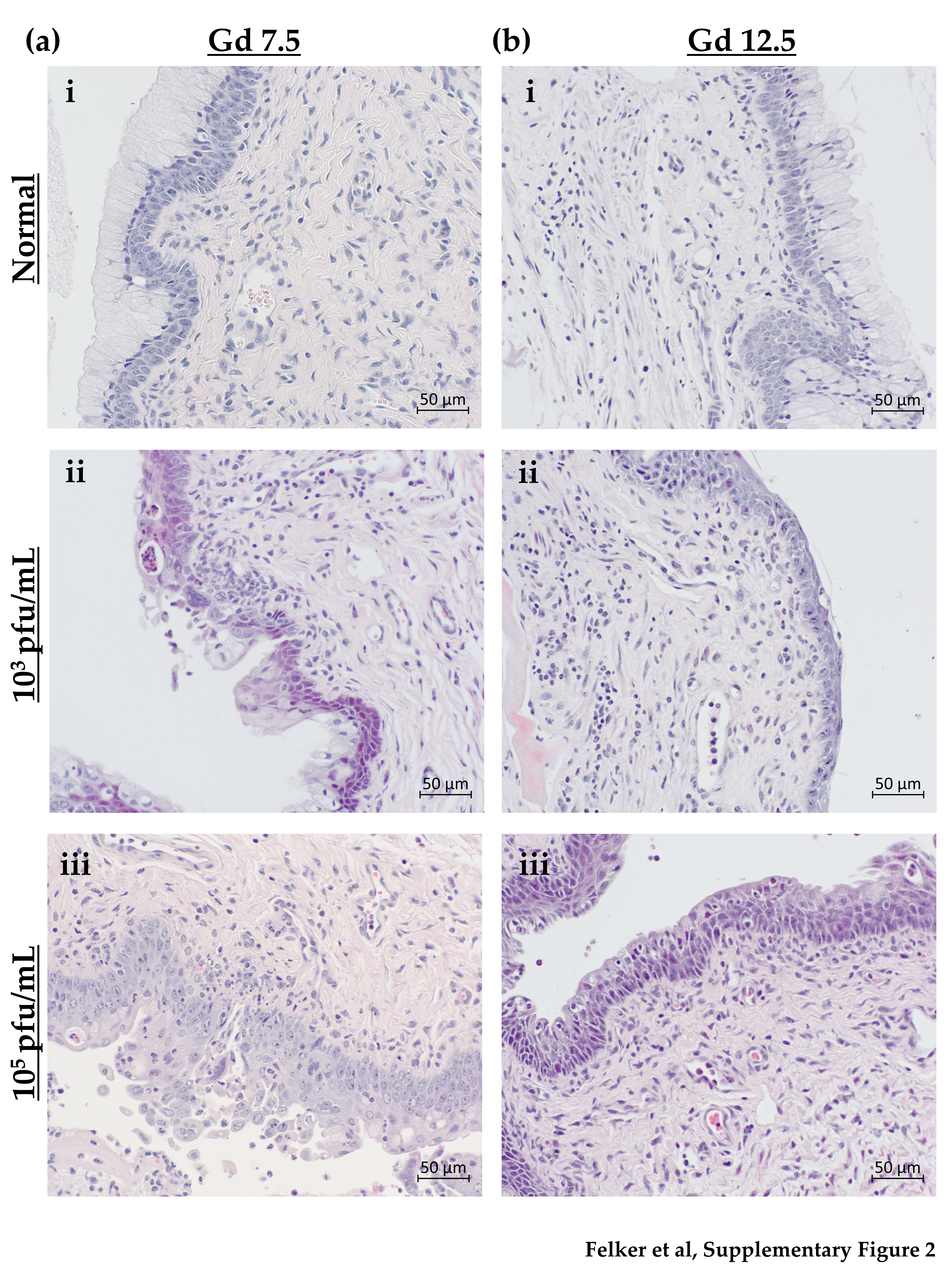

Supplement: Supplementary file 1 [file viruses-13-01929-s001.zip › Felker et al Supp Figure 2.tif]
